# Measuring implementation fidelity in a cluster-randomized pragmatic trial: development and use of a quantitative multi-component approach

**DOI:** 10.1186/s13063-022-06002-8

**Published:** 2022-01-15

**Authors:** Miranda B. Olson, Ellen M. McCreedy, Rosa R. Baier, Renée R. Shield, Esme E. Zediker, Rebecca Uth, Kali S. Thomas, Vincent Mor, Roee Gutman, James L. Rudolph

**Affiliations:** 1 Center for Long-Term Care Quality & Innovation, Brown University School of Public Health, 121 South Main St., Providence, RI 02912 USA; 2 Center for Gerontology & Healthcare Research, Brown University School of Public Health, 121 South Main St., Providence, RI 02912 USA; 3 Department of Health Services, Policy & Practice, Brown University School of Public Health, 121 South Main St., Providence, RI 02912 USA; 4 US Department of Veterans Affairs Medical Center, 830 Chalkstone Ave., Providence, RI 02908 USA; 5 Department of Biostatistics, Brown University School of Public Health, 121 South Main St., Providence, RI 02912 USA

**Keywords:** Implementation, Fidelity, Adherence, Pragmatic trial, Nursing home, Dementia

## Abstract

**Background:**

In pragmatic trials, on-site partners, rather than researchers, lead intervention delivery, which may result in implementation variation. There is a need to quantitatively measure this variation. Applying the Framework for Implementation Fidelity (FIF), we develop an approach for measuring variability in site-level implementation fidelity. This approach is then applied to measure site-level fidelity in a cluster-randomized pragmatic trial of Music & Memory^SM^ (M&M), a personalized music intervention targeting agitated behaviors in residents living with dementia, in US nursing homes (NHs).

**Methods:**

Intervention NHs (*N* = 27) implemented M&M using a standardized manual, utilizing provided staff trainings and iPods for participating residents. Quantitative implementation data, including iPod metadata (i.e., song title, duration, number of plays), were collected during baseline, 4-month, and 8-month site visits. Three researchers developed four FIF adherence dimension scores. For Details of Content, we independently reviewed the implementation manual and reached consensus on six core M&M components. Coverage was the total number of residents exposed to the music at each NH. Frequency was the percent of participating residents in each NH exposed to M&M at least weekly. Duration was the median minutes of music received per resident day exposed. Data elements were scaled and summed to generate dimension-level NH scores, which were then summed to create a Composite adherence score. NHs were grouped by tercile (low-, medium-, high-fidelity).

**Results:**

The 27 NHs differed in size, resident composition, and publicly reported quality rating. The Composite score demonstrated significant variation across NHs, ranging from 4.0 to 12.0 [8.0, standard deviation (SD) 2.1]. Scaled dimension scores were significantly correlated with the Composite score. However, dimension scores were not highly correlated with each other; for example, the correlation of the Details of Content score with Coverage was *τ*_b_ = 0.11 (*p* = 0.59) and with Duration was *τ*_b_ = − 0.05 (*p* = 0.78). The Composite score correlated with CMS quality star rating and presence of an Alzheimer’s unit, suggesting face validity.

**Conclusions:**

Guided by the FIF, we developed and used an approach to quantitatively measure overall site-level fidelity in a multi-site pragmatic trial. Future pragmatic trials, particularly in the long-term care environment, may benefit from this approach.

**Trial registration:**

Clinicaltrials.gov NCT03821844. Registered on 30 January 2019, https://clinicaltrials.gov/ct2/show/NCT03821844.

**Supplementary Information:**

The online version contains supplementary material available at 10.1186/s13063-022-06002-8.

## Background

In recent years, researchers have proposed embedded pragmatic clinical trials (ePCTs) as a potential solution for the persistent gap between efficacy and effectiveness data [1]. While many non-pharmaceutical interventions have proven beneficial in traditional randomized, clinical trials (RCTs), most of these interventions have not been adapted for or tested in real-world settings [1, 2]. ePCT designs minimize researcher involvement in implementation efforts and use pre-existing processes, such as routinely collected data, to capture study outcomes [3]. Unlike traditional RCTs, ePCTs allow for implementation processes and clinical outcomes to be studied simultaneously [4]. This design offers the possibility of accelerating the generation of real-world effectiveness evidence [5].

Because intervention delivery in ePCTs is not led by researchers as in traditional RCTs, capturing variation in implementation is critical for understanding outcome results. One method for capturing this information is to assess implementation fidelity, the degree to which interventions are implemented as intended [6]. Studies show that fidelity is an important potential moderator between intervention delivery and intended outcomes [7–9]. Previous ePCTs have included fidelity analyses [10]. However, many of these trials relied on a simple, exposed versus unexposed quantitative measure for fidelity, such as whether or not a participant viewed the intervention video [11, 12]. Other studies, applying a more complex definition of implementation fidelity, develop an intervention-specific measurement tool without the guidance of an a priori theoretical framework [13, 14]. Neither of these measurement approaches capture a holistic view of fidelity, especially considering the complexity of many interventions. Other studies, involving a multifaceted definition of implementation fidelity, present mostly qualitative analyses [15]. These analyses remain limited in their usefulness for interpreting the effect of fidelity on quantitative clinical outcomes. There is a need for a method to quantitatively measure variability of implementation fidelity in ePCTs examining complex interventions.

We implemented a cluster-randomized ePCT designed to test the effectiveness of a complex music intervention in the long-term care environment for people living with Alzheimer’s Disease and Related Dementias (ADRD). Currently 47% of United States (US) nursing home (NH) residents live with ADRD, a disease of progressive cognitive dysfunction leading to functional impairments and behavioral changes that require increased supervision over time [16], and the prevalence of Alzheimer’s disease is predicted to double by 2060 in the US [17]. Agitated behaviors are common in this population [18] and are often addressed via prescription of psychoactive medications associated with dangerous adverse effects including increased risk of falls and death [19]. With this population of NH residents rapidly increasing, alternative strategies for managing agitated behaviors are desperately needed.

Personalized music is one alternative strategy which may hold promise to safely manage agitated behaviors in the NH setting. One popular program, Music and Memory^SM^ (M&M), focuses on using individualized music when a person living with ADRD is prone to agitated behaviors, such as during specific times of day or care actions (e.g., dressing, grooming, bathing) [20]. Research shows that music enjoyed earlier in life may be stored in an area of the brain affected later in the dementia disease course [21]. M&M leverages the potential of this early preferred music to generate positive emotions in an individual with ADRD, temporarily alleviating agitated behaviors. Many NHs have already adopted M&M without effectiveness evidence or proven implementation guidance.

Only three evaluations have previously examined the effectiveness of M&M. So far, there is one relatively small trial of this intervention (*N* = 59) [22]. There have also been two larger evaluations of M&M, as administered in a real-world setting. The first is a quasi-experimental study [23], and the second is a pre-post evaluation [24]. These evaluations have resulted in mixed effectiveness evidence regarding the M&M program, motivating this ePCT. Indeed, the largest M&M trial to date cites low adherence with high variability in implementation as the rationale for overall null results [22].

As the demand for ePCTs in NHs increases, effectively capturing variation in implementation fidelity is critical for interpreting generated results, particularly in multi-site trials [2]. We implemented an ePCT designed to test the effect of M&M on agitation among people living with ADRD who reside in geographically and socio-demographically diverse long-term NHs (Clinicaltrials.gov ID: NCT03821844) [25]. This paper presents an approach, guided by a theoretical framework, for measuring site-level multi-dimensional implementation adherence to quantitatively describe variability in implementation fidelity across participating US NHs.

## Methods

### Study design and participants

Music & MEmory: A Pragmatic TRial for Nursing Home Residents With Alzheimer’s Disease (METRIcAL) was a parallel, cluster-randomized ePCT of M&M. This study was designed as a pilot hybrid type 2 trial [26], which simultaneously evaluates intervention effectiveness and implementation [4, 27]. The details of the trial are described elsewhere (Clinicaltrials.gov ID: NCT03821844) [25]. The trial was conducted in 54 US NHs (*N* = 27 intervention, *N* = 27 control) from four NH corporations that differed in size, geographical location, residents’ racial composition, and organizational structure. Potentially eligible NHs had at least 20 residents who were long-stay (90 of the last 100 days spent in the NH), had a dementia diagnosis, and were not completely deaf. Within each corporation, researchers used NH characteristics data to identify potentially eligible NHs. Corporate leads were then allowed to exclude NHs known to have pre-existing challenges that may prevent participation, such as recent poor performance surveys or unstable leadership. Depending on the corporation, either researchers or corporate leadership offered the selected NHs the opportunity to participate in the study. Administrators at NHs which decided to opt-in signed a letter of intent describing participation and randomization requirements prior to study initiation. At each intervention facility, the personalized music intervention was implemented via an embedded approach, using a team approach led by a designated NH champion. Recruitment and randomization were completed in February 2019 with the trial running from June 2019 through January 2020. This reporting of the study implementation follows the Standards for Reporting Implementation Studies (StaRI) guidelines as well as the Template for Intervention Description and Replication (TIDieR) guidelines (see Additional File 1). This was deemed a minimal risk study by the Institutional Review Board, which issued a waiver of individual consent (#1705001793).

### Framework for Implementation Fidelity

**Fig. 1:**
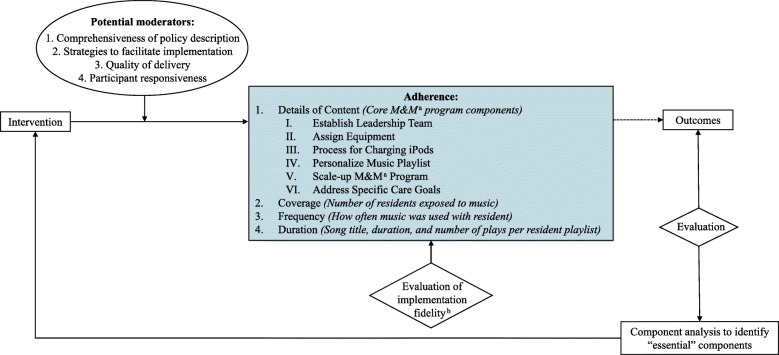
An overview of the conceptual Framework for Implementation Fidelity as applied to the METRIcAL study. For each adherence dimension presented in the original model, the study-specific definition and identified data elements are summarized

Three investigators (MO, EM, and JR) applied the Framework for Implementation Fidelity (FIF) to describe adherence as the primary measurement of implementation fidelity (see Fig. 1) [28]. The FIF presents four dimensions of adherence: (1) Details of Content—*what* was delivered to participating residents in each NH; (2) Coverage—*how many* residents received the intervention; (3) Frequency—*how often* the intervention was delivered to these residents; and (4) Duration—*how long* the intervention was delivered in each interaction [28]. We apply these four adherence dimensions to our pragmatic trial of M&M in NHs to qualify and quantify fidelity by aligning with best-practice implementation protocols.

### Description of materials

In the M&M program, each resident is assigned a personal music device (e.g., iPod) and earphones. NH staff identified music preferred by the resident as a young adult and then loaded this preferred music onto the resident’s device. NH staff then initiated music use with the resident at times of day when agitated behaviors are likely or at early signs of agitation [4]. The recommended dose is 30 min of music per day with each resident.

To promote consistency in intervention delivery and scale-up across the participating NHs, a guide for M&M implementation was developed during the pilot phase of this study. This implementation guide provides participating NHs with concise, step-by-step guidance on key aspects of the M&M program.

Participating NHs received two types of staff training: (1) standard M&M Certification Training and (2) an in-person training developed specifically for this trial. The standard M&M Certification Training consists of two 90-min live webinars focused on teaching staff how to pilot and scale-up the M&M program in their NH [29]. As a supplement to this standard training, corporate leadership and study consultants administered an in-person, study-specific training. This in-person training provided a detailed walk-through of the M&M implementation guide and allowed for thorough discussion and Q&A sessions, shared learning between staff, and expanded implementation suggestions, particularly regarding the process for developing an individualized music playlist. The following staff from each NH were encouraged to attend these in-person sessions: NH administrator, director of nursing, activities director, social work director, and a nurse manager. For each NH, one attendee was identified as the M&M champion. In addition to these preparatory trainings, corporate leadership and investigators co-led monthly coaching calls with each NH to report progress, discuss challenges, and celebrate achievements for the duration of the study.

After completing these staff trainings, each participating NH was provided with 20 personal music players, 20 charging cables, iTunes gift cards, two multi-port charging stations, three small speakers, and one computer tablet with the iTunes application downloaded.

### Data sources

Several sources of implementation data were collected during the 8-month trial, including (1) interviews with NH staff, (2) observations by trained data collectors during site visits, (3) metadata downloaded from the music players, (4) forms completed by NH staff at time of first music use, (5) research staff impressions of each NH, and (6) NH characteristics data from the publicly-available Brown University website LTCFocus.org [30].

#### NH staff interviews

Ten trained data collectors visited each participating NH three times during the course of the trial: at baseline, 4 months (mid-implementation), and 8 months (full-implementation). Site visits were conducted over 2 or 3 days and included interviews with NH staff about each resident’s experience while listening to music.

#### Observation tool

Following each site visit, the same data collectors completed a structured summary observation form describing how engaged the site was in the M&M program and different logistical aspects of setup, including individualized labeling of devices and device charging procedures.

#### iPod metadata

Using iTunes interface, trained data collectors assisted NH staff in downloading music play data for each resident. Music play data included the name and artist for every song on the resident’s playlist, as well as the duration of each song and the number of times it was played.

#### Initial Use Form

For each resident exposed to the program, staff completed an Initial Use Form. This form was completed by the M&M champion at each NH. The Initial Use Form describes why the resident was chosen for the music program, how and when the resident’s personalized music was identified, and how the resident reacted to the music.

#### Research staff ratings

Two research assistants who worked closely with NH staff throughout the study independently scored the engagement of the administrator, nursing staff, and program champion at each NH as low (1), medium (2), or high (3) engagement. The research assistants then met to discuss each score. Consensus was reached, producing three engagement scores for each NH.

#### LTCFocus.org

Partially funded by the National Institute on Aging (1P01AG027296), LTCFocus.org is a website and dataset hosted by the Shaping Long Term Care in America Project at Brown University [30]. This source incorporates data from multiple datasets, including the Minimum Data Set (MDS), Certification and Survey Provider Enhanced Reporting (CASPER) system, area resource file, and residential history file. LTCFocus.org was used to capture NH characteristics in advance of and throughout the study period.

### Dimensions of adherence

#### Details of Content

Capturing *what* was delivered to residents, Details of Content is the most complex and intervention-specific dimension. In a complex intervention, such as M&M, many components combine to form the intended intervention. However, only some of these components are essential to the primary function of the intervention. These core components may be thought of as the “active ingredients” of the intervention and are actions that NHs must complete to provide the M&M intervention, as intended by the intervention’s creators [28].

To identify the core components of the M&M program, three investigators (MO, EM, and JR) first independently reviewed the M&M implementation manual and then met to discuss the core components each investigator had identified. This process was repeated until the investigators reached consensus, with at least two of the three investigators agreeing on the core components. Two study consultants reviewed and, without suggesting significant edits, approved the final list of M&M core components. Through this process, we identified six core components of the M&M program (Table 2).

I. *Establish Leadership Team* was operationalized as the perceived engagement of NH staff in M&M implementation, as engagement seemed to be associated with on-site leadership. The three engagement scores from the Research Staff Ratings were summed for each NH to create a facility-level engagement score.

II. *Assign Equipment* was operationalized as whether the M&M equipment was properly labeled for individual residents. The representative data element was the Observational Tool question, “Was each iPod and headphone assigned to an individual resident?” If the data collector observed each iPod and headphone set labeled for an individual resident, the NH receives a high (1) score. If not all of the equipment was labeled properly, the NH receives a low (0) score.

III. *Process for Charging iPods* was operationalized as whether the data collector observed a process for charging equipment at either the midpoint or final data collection. The representative data element was the Observational Tool question, “Did you observe processes for charging iPods?” If the data collector observed processes, the NH receives a high (1) score. If no processes were observed, the NH receives a low (0) score.

IV. *Personalize Music Playlist* was operationalized as the average percent of unique songs on each resident’s playlist in a given NH. Using the iPod metadata, the song titles from each resident’s playlist were compared to the song titles from the other resident playlists in that NH. The songs not found on the other playlists were considered unique to that resident. The percent of unique songs on each playlist were then averaged for each NH to generate a facility-level measure.

V. *Scale-up M&M Program* was operationalized as the difference between the number of residents who were exposed at the midpoint and final data collection visits, compared to the expected numbers at these two timepoints, as established by the M&M implementation guide (eight and 15 residents, respectively). The Initial Use Form was used to identify each resident’s initial date of exposure. For each NH, the number of residents exposed to M&M at midpoint and final data collection were subtracted from the respective expected number of residents. The absolute values for these differences were then summed to generate a facility-level score.

VI. *Address Specific Care Goals* was operationalized as the percent of residents in a given NH who were selected for the program to address a specific goal of care. The Initial Use Form indicates whether a resident was selected for M&M to address any of the following clinical care goals: acute illness, verbal and physical agitation, bathing, eating, administration of medications, reduction of medications, morning care, pain, and rejection of care. For each NH, the percentage of residents participating in the trial who were selected to address any of these clinical goals was calculated.

#### Coverage

Coverage is defined as the number of residents exposed to the intervention. In this study, completion of an Initial Use Form indicates that a specific resident was assigned an iPod and participated in the M&M program. Therefore, Coverage was calculated as the number of Initial Use Forms completed at each NH during the study period. The maximum number of participating residents was limited by the number of provided music players.

#### Frequency

Frequency is defined as the number of interactions participating residents have with the intervention. However, frequency was operationalized as the percentage of participating residents exposed to the music at least once per week by nursing staff. The NH Staff Interviews included the following multiple-choice question: “In the past two weeks, how often have you used the music with the resident?” Based on the response distribution, frequent use of the M&M program was defined as weekly music use. Therefore, Frequency was calculated as the percentage of participating residents exposed to music at least once per week in each NH, as reported by NH staff.

#### Duration

This was defined as the amount, or length, of intervention provided in each interaction. Duration was operationalized as the median minutes of music received per resident day exposed, averaged over all the residents in a given facility. Using the length of each song and number of plays from the iPod metadata, researchers approximated the total minutes of exposure each resident received during the study period. Exposed days were calculated as the number of days the resident had access to the music, based on the Initial Use Form start date and end of follow-up date. Minutes per exposed day (per resident) is calculated as the total minutes divided by exposed days.

### Resident composition and NH characteristics

Exploratory analyses included several resident composition descriptors including resident gender, race, antipsychotic use, cognitive impairment, and activities of daily living (ADL) score [30]. The following NH characteristics were also included: number of beds, presence of specialized Alzheimer’s unit, Centers for Medicare & Medicaid Services (CMS) 5-Star Quality Rating [31], payor status, registered nurse hours per resident day, and licensed practical nurse hours per resident day.

### Analyses

To construct a Details of Content score, responses for each M&M core component were scaled to low (1), medium (2), and high (3) adherence based on data distributions and theoretical significance. These scaled scores were then summed to create an overall Details of Content score. To construct a Composite adherence score, the facility-level score for each FIF adherence dimension was then scaled low (1), medium (2), and high (3) by using the terciles of the corresponding data distributions. These scaled FIF dimension scores were summed to generate a composite adherence score. The raw composite score was then scaled to low (1), medium (2), or high (3) adherence based on the composite score distribution. This scaled Composite score identifies high-, medium-, and low-fidelity NHs.

Fidelity in the FIF dimensions and overall were assessed via descriptive statistics. Kendall’s tau-b (*τ*_b_) correlations between the six M&M core components as well as between the FIF dimensions scores and Composite scores are reported. Baseline facility-level characteristics were compared for the high- and low-fidelity NHs, as identified by the Composite adherence score.

## Results

On average, the 27 implementation NHs were large [101.5 beds, standard deviation (SD) 42.3] with primary Medicaid payment source (58.8%, SD 25.6) and above average quality rating (3.5 Medicare star rating, SD 1.4; range 1–5; higher ratings indicate higher quality) (Table 1). These NHs on average primarily housed female (60%) residents with significant moderate-to-severe cognitive impairment (dementia = 64.1%, SD 11.8) and functional impairment (16.7 Activities of Daily Living score, SD 1.7; range 7–28; higher scores indicate worse function).

**Table 1:** Characteristics of nursing homes implementing the Music & Memory program

	Intervention Nursing Homes (*n* = 27)
	Mean (SD)
**Resident composition and acuity**^**a**^	
Female (%)	65.4 (10.9)
Black (%)	22.3 (25.7)
Moderate or severe cognitive impairment (%)	64.1 (11.8)
Any antipsychotic use (%)	17.9 (8.6)
Total ADL score^b^	16.7 (1.7)
**Nursing home characteristics**	
Total beds (#)	101.5 (42.3)
Alzheimer’s unit (%)	23.1 (43.0)
CMS^c^ 5-Star Quality Rating^d^	3.5 (1.4)
Medicaid as primary payer (%)^e^	58.8 (25.6)
Medicare as primary payer (%)	11.2 (7.0)
Other/Self-pay (%)	30.1 (26.4)
Registered nurse hours per resident day (#)	0.3 (0.2)
Licensed practical nurse hours per resident day (#)	0.9 (0.3)

^a^All data represent facility-level characteristics at baseline

^b^Describes ability of resident to perform activities of daily living (ADLs). Higher scores indicate more dependence on staff (range 7–28) [39].

^c^Centers for Medicare & Medicaid Services

^d^Score ranges from 1 to 5 stars, with five stars indicating the highest quality nursing homes [31]

^e^In the US, Medicare is a health insurance program for citizens aged 65 years and older. Medicare does not provide coverage for long-term care but does provide payment for qualified short-term rehabilitation after hospitalization. Medicaid is a health program for qualified people of all ages related to disability, income, or disease. Medicaid does provide long-term care, and the payment is generally well below that of Medicare. From the nursing home perspective, Medicare is a payor of choice [40]

### Dimensions of implementation fidelity

#### Details of Content

The *Establish Leadership Team* score ranges from 3.0 to 9.0 (6.4, SD 2.1), identifying nine low NHs (range 3.0–5.0) and eight high NHs (range 8.0–9.0) for this M&M component (Table 2). The dichotomized *Assign Equipment* score (range − 1.0 to 2.0) identifies 7 NHs with a high score (2.0). Similarly, the dichotomized *Process for Charging iPods* score (range − 2.0 to 2.0) identifies 12 high NHs (range 0.0–2.0). The *Personalize Music Playlist* score ranges from 0.0 to 1.0 (mean 0.5, SD 0.3) and identifies nine low NHs (range 0.0–0.1) and nine high NHs (range 0.3–1.0). The *Scale-up M&M Program* score ranges from 0.0 to 6.0 (mean 2.2, SD 1.7), identifying nine low NHs (range 3.0–6.0) and 11 high NHs (range 0.0–1.0). The *Address Specific Care Goals* score ranges from 0.0 to 0.9 (mean 0.5, SD 0.3) with nine low NHs (range 0.0–0.3) and nine high NHs (range 0.7–0.9).

**Table 2:** Details of Content: core components of Music & Memory (M&M) and corresponding data elements

M&M core component	Data element	Data source	distribution
I. Establish Leadership Team	Administrator, Nursing, and Site Champion receptiveness to program	Research Staff Ratings	Range: 3.0–9.0 Mean (SD): 6.4 (2.1)
II. Assign Equipment	Was each iPod and headphone assigned to an individual resident?	Observation Tool	Dichotomized Frequency: 7, 20
III. Process for Charging iPods	Did you observe processes for charging iPods?	Observation Tool	Dichotomized Frequency: 15, 12
IV. Personalize Music Playlist	Uniqueness of songs on individual playlists based on song title	iPod Metadata	Range: 0.0–1.0 Mean (SD): 0.5 (0.3)
V. Scale-up M&M Program	Number of residents exposed at midpoint and end of follow-up	Initial Use Form	Range: 0.0–6.0 Mean (SD): 2.2 (1.7)
VI. Address Specific Care Goals	Why did you choose this resident to participate in the program?	Initial Use Form	Range: 0.0–0.9 Mean (SD): 0.5 (0.3)

The six scaled M&M core component scores were not highly correlated with one another (Table 3). The *Establish Leadership Team* and *Scale-up M&M Program* scores were significantly correlated (*τ*_b_ = 0.43, *p* < 0.05) as well as the *Process for Charging iPods* and *Address Specific Care Goals* scores (*τ*_b_ = 0.43, *p* < 0.05). The remaining correlations indicate relatively weak associations between the M&M core components (Table 3).

**Table 3:** Correlation coefficients (*τ*_b_) and significance values between scaled Music and Memory (M&M) core component scores

	I. Leadership team	II. Assign equipment	III. Charging iPods	IV. Personalize playlist	V. Scale-up M&M	VI. Specific care goals
**I. Leadership team**	1.00					
**II. Assign equipment**	−0.24	1.00				
**III. Charging iPods**	−0.25	0.29	1.00			
**IV. Personalize playlist**	0.23	−0.21	0.11	1.00		
**V. Scale-up M&M**	0.39*	−0.24	0.05	−0.11	1.00	
**VI. Specific care goals**	−0.06	0.10	0.44*	−0.11	0.21	1.00

*p-value < 0.05

When summed, the scaled M&M core component scores generate a Details of Content score ranging from 7.0 to 14.0 (mean 9.6, SD 1.0) with the following tercile ranges: low (7.0–8.0), medium (9.0–10.0), and high (11.0–14.0) (Table 4).

**Table 4:** Framework for Implementation Fidelity (FIF) dimension scores, Composite adherence score, and corresponding data elements

Adherence dimension	Data element(s)	Data source(s)	Construction	Distribution	High range NHs
1. Details of content	M&M core components data elements	Displayed Table 2	Data elements were scaled (1–3) and summed	Range: 7.0–14.0 Mean (SD): 9.6 (1.0)	11–14 (9 NHs)
2. Coverage	Number of residents exposed to M&M program	Initial Use Form	Number of completed Initial Use Forms	Range: 4.0–15.0 Mean (SD): 12.4 (3.5)	15 (9 NHs)
3. Frequency	In the past 2 weeks, how often have you used the music with the resident?	NH Staff Interview	% residents in NH with nurses using music at least once per week	Range: 0.0–1.0 Mean (SD): 0.4 (0.3)	0.67–1.0 (9 NHs)
4. Duration	Song title, duration, and total number of plays per resident playlist	iPod Metadata	Median minutes of played music per resident day exposed	Range: 0.0–99.0 Mean (SD): 29.8 (25.9)	30–99 (9 NHs)
**Composite adherence score**	Adherence Dimensions data elements	Adherence Dimensions Data Sources	Details of content, Coverage, Frequency, and Duration data elements scaled (1–3) and summed	Range: 4.0–12.0 Mean (SD): 8.0 (2.1)	9–12 (10 NHs)

#### Coverage

The Coverage score ranges from 4.0 exposed residents to 15.0 (mean 12.4, SD 3.5) (Table 4). The Coverage score identifies nine NHs in each of the following terciles: low (4.0–13.0), medium (14.0), and high (15.0).

#### Frequency

The Frequency score ranges from 0.0 to 1.0 and was normally distributed, with high variability between NH scores (mean 0.4, SD 0.3) (Table 4). This score identifies nine NHs in each of the following tercile ranges: low (0.0–0.2), medium (0.3–0.6), and high (0.7–1.0).

#### Duration

The Duration score ranges from 0.0 to 99.0 median minutes per resident day exposed (mean 29.8, SD 25.9), with increased playtime indicating the resident received more music (Table 4). This score identifies nine NHs in each of the following tercile ranges: low (0.0–13.0), medium (16.0–28.0), and high (34.0–99.0).

### Composite adherence score

Each scaled FIF dimension score had an equal distribution of NHs across the low (1), medium (2), and high (3) categories (nine NHs per category) (Table 4). These scaled dimension scores were summed to generate a Composite adherence score which ranges from 4.0 to 12.0 (mean 8.0, SD 2.1). This Composite score identifies eight low NHs (4.0–7.0), nine medium NHs (8.0), and ten high NHs (9.0–12.0).

Because the construction of the Composite adherence score is based on the FIF dimensions, the scaled FIF dimension scores were significantly correlated with the Composite score, with the Frequency score displaying the strongest association (*τ*_b_ = 0.58, *p* < 0.01) and the Duration score the weakest association (*τ*_b_ = 0.40, *p* < 0.05) (Table 5). However, dimension scores were not highly correlated with each other. For example, the correlation between the Details of Content and the Coverage scores was *τ*_b_ = 0.11 (*p* = 0.59), and the correlation between the Details of Content and the Duration scores was *τ*_b_ = − 0.05 (*p* = 0.78) (Table 5). The Frequency score also displays a weak association with the Duration (*τ*_b_ = 0.05, *p* = 0.81) and Coverage (*τ*_b_ = 0.14, *p* = 0.42) scores. The Details of Content and Frequency scores exhibit the strongest association between the scaled dimension scores (τ_b_ = 0.43, *p* < 0.05) (Table 5).

**Table 5:** Correlation coefficients (*τ*_b_) and significance values between scaled FIF dimensions scores and Composite adherence scores

	Details of Content	Coverage	Frequency	Duration	Composite
**Details of Content**	1.00				
**Coverage**	0.10 (0.59)	1.00			
**Frequency**	0.43 (0.01)	0.14 (0.42)	1.00		
**Duration**	−0.05 (0.78)	0.35 (0.05)	0.05 (0.81)	1.00	
**Composite**	0.52 (0.00)	0.50 (0.00)	0.58 (0.00)	0.40 (0.02)	1.00

*p-values presented in parentheses

### Comparison of high- and low-fidelity NHs

Using the Composite adherence score, ten high-fidelity NHs and eight low-fidelity NHs were identified (Table 6). On average, high-fidelity NHs were smaller (mean 89.0 beds, SD 20.7) than the low-fidelity NHs (mean 107.4 beds, SD 28.6) and tended to have a higher percentage of residents with moderate or severe cognitive impairment (65.9%, SD 16.5%) than low-fidelity facilities (59.8%, SD 10.1%). The high-fidelity NHs had better quality ratings on average (mean 3.6 Medicare star rating, SD 1.4) than the low-fidelity NHs (mean 2.0 Medicare star rating, SD 0.5) and were more likely to have an Alzheimer’s unit (22.2%, SD 44.1%) than the low-fidelity NHs (12.5%, SD 35.4%). Additionally, high-fidelity NHs have more antipsychotic use on average at baseline (20.6%, SD 10.1%) than low-fidelity NHs (16.4%, SD 10.1%).

**Table 6:** Characteristics of low- and high-fidelity nursing homes, as identified by the Composite adherence score

	Low-fidelity nursing homes (*n* = 8)	High-fidelity nursing homes (*n* = 10)
	Mean (SD)	Mean (SD)
**Resident composition and acuity**^**a**^		
Female (%)	67.5 (12.6)	65.0 (8.0)
Black (%)	24.7 (27.1)	23.1 (20.0)
Moderate or severe cognitive impairment (%)	59.8 (10.1)	65.9 (16.5)
Any antipsychotic use (%)	16.4 (10.1)	20.6 (10.1)
Total ADL score^b^	16.4 (1.4)	17.1 (1.9)
**Nursing home characteristics**		
Total beds (#)	107.4 (28.6)	89.0 (20.7)
Alzheimer’s unit (%)	12.5 (35.4)	22.2 (44.1)
CMS^c^ 5-Star Quality Rating^d^	2.0 (0.5)	3.6 (1.4)
Medicaid as primary payer (%)^e^	63.0 (22.2)	67.6 (15.2)
Medicare as primary payer (%)	9.6 (4.3)	13.8 (8.9)
Other/self-pay (%)	27.5 (21.6)	18.7 (13.8)
Registered nurse hours per resident day (#)	0.3 (0.2)	0.3 (0.2)
Licensed practical nurse hours per resident day (#)	0.8 (0.3)	0.9 (0.4)

^a^All data represent facility-level characteristics at baseline

^b^Describes ability of resident to perform activities of daily living (ADLs). Higher scores indicate more dependence on staff [32].

^c^Centers for Medicare & Medicaid Services

^d^Score ranges from 1 to 5 stars, with five stars indicating the highest quality nursing homes [31].

^e^In the US, Medicare is a health insurance program for citizens aged 65 years and older. Medicare does not provide coverage for long-term care but does provide payment for qualified short-term rehabilitation after hospitalization. Medicaid is a health program for qualified people of all ages related to disability, income, or disease. Medicaid does provide long-term care, and the payment is generally well below that of Medicare. From the nursing home perspective, Medicare is a payor of choice [33]

## Discussion

Using the FIF conceptual model, we describe a replicable and feasible approach for quantitatively describing the structure, function, and essential dimensions of implementation fidelity for a cluster-randomized ePCT of a complex intervention. The four FIF adherence dimension scores—Details of Content, Coverage, Frequency, and Duration—are not highly correlated, highlighting the importance of considering different dimensions of overall implementation fidelity. Initial tests of face validity are promising, as the Composite adherence score is correlated with aspects of NH quality which are likely to affect implementation including CMS quality star rating and presence of an Alzheimer’s specialty unit [15–18]. This study suggests that NHs may perform highly in one adherence domain, but not others. The Composite score successfully captures variability in overall implementation fidelity, indicating that it is feasible and useful to develop a composite measure of implementation fidelity to distinguish facility-level adherence to an intervention implementation protocol. These results have implications for measurement of implementation fidelity in cluster-randomized ePCTs, particularly in the long-term care environment.

Given the findings of previous studies examining the M&M program [22], METRIcAL focused on thoroughly training participating NH staff, specifically using the implementation guide tested and revised during the pilot phase of this trial, as well as providing additional support via corporate leads and our research team throughout the study duration. This study identifies six core components essential for achieving complete fidelity, as defined by developers, in implementing the M&M program. As expected in an ePCT, facility-level variation in implementation fidelity was observed for each core component, despite best efforts to standardize implementation across participating NHs. In general, NHs did not ubiquitously score as high fidelity across the core components. Rather, each NH tended to have high fidelity for some components and medium or low fidelity for the others. These results highlight the challenges of conducting an ePCT examining a complex intervention. However, the quantitative evaluation of component-level implementation fidelity was feasible because of our rigorous, multimodal data collection approach.

There are precedents for using the FIF as a guiding framework for measuring implementation adherence for this trial design as well as in this care environment [34]. The Pragmatic Trial of Video Education in Nursing Homes (PROVEN), a cluster-randomized ePCT examining the effect of advanced care planning for residents living with ADRD, conducted mixed-methods analyses of implementation fidelity [11]. The PROVEN study presented structured qualitative interview data analyses within the FIF adherence dimensions to contextualize high and low NH fidelity, as defined by a single quantitative fidelity measure (exposed versus unexposed) [15]. Palmer et al. provided critical lessons learned across the FIF dimensions, but with only one quantitative measure, they did not produce a multi-dimensional quantitative measure of implementation fidelity. However, PROVEN did show that understanding implementation fidelity is critical for properly interpreting the results of pragmatic trials. In the Netherlands, a cluster-randomized ePCT of the Geriatric Care Model, a home-based chronic care management program, also utilized the FIF to measure implementation adherence [35]. Specific questions addressing the four adherence dimensions were collected mid-implementation and after the study concluded, while two moderating factors were assessed after study completion [36]. However, this study of the Geriatric Care Model was limited to qualitative data. Other ePCTs have used the FIF as a guiding framework for implementation fidelity analyses, but none have produced a composite measure of adherence [36–38].

The FIF has also been used as a guiding framework for implementation analyses in RCTs studying complex care interventions in the elderly population [39, 38]. A notable process evaluation conducted in Norway was the only identified study which constructed a quantitative composite measurement of implementation adherence guided by the FIF [40]. This mixed-methods study accompanied an RCT examining an intervention to promote psychosocial well-being in stroke survivors with and without aphasia during early rehabilitation. This thorough study examines the separate dimensions of implementation adherence, potential modifiers, and presents a quantitative composite measure [40]. While the construction of the composite measure uses a similar approach as the one presented in this paper, Bragstad et al. use theoretically significant cutoff points to define high, medium, and low fidelity. Therefore, the composite measure in this study is used to establish, “that four out of five interventions were implemented with high fidelity,” and not to describe variability in overall implementation fidelity [40].

### Study limitations

This study is limited to program implementation in 27 US NHs. Although diverse, this sample of NHs limits the generalizability of the presented results as well as the statistical significance of these analyses. Trained data collectors gathered the included implementation data through interviews, observation, and reporting. Each of these data collection methods present different biases and measurement scales. For example, observer and recall biases may have affected the NH staff interview responses, particularly as participants and data collectors were not blinded to the intervention. However, through rigorous training and standardized data collection tools, the potential for bias was minimized wherever possible for all data sources. Furthermore, when selecting representative data elements, we retroactively chose data elements that “best fit” the theoretically significant M&M core components and FIF adherence dimensions. Given the secondary use of some data elements, there may be discrepancy between the intended variable and selected element. Finally, potential moderators included in the FIF conceptual framework (see Fig. 1) likely affect the composite adherence score, but these were beyond the scope of this paper.

## Conclusions

Expanding on previous literature, our team develops and applies an approach to quantitatively measure site-level multi-component implementation adherence for an ePCT using the FIF as a guiding framework. The resulting dimension and Composite adherence scores capture significant variability in implementation fidelity across participating NHs with varied characteristics. By capturing variability in implementation fidelity across the core components of the intervention, the Details of Content dimension allows the presented approach to be adapted for a variety of complex interventions. This approach also allows for differentiation of implementation fidelity across participating sites at various levels: intervention components, dimensions of adherence, and overall fidelity. As indicated by the low association of the dimension scores, these aspects of implementation fidelity are distinct. Therefore, each quantitative sub-score provides a separate opportunity to study the moderating effects of implementation fidelity on primary and secondary clinical outcomes. Additionally, these sub-scores may be used to investigate which intervention components and aspects of adherence are most significant in achieving the desired intervention outcomes. The presented approach may be beneficial for future ePCTs to quantitatively examine the effect of implementation fidelity on study outcomes. Next steps for this work include using the generated dimension and Composite adherence scores to examine the effect of adherence on primary and secondary study outcomes. These analyses will also be extended to include the effect of the FIF potential moderators.

## Supplementary Information

**Additional file 1.** Portable Document Format (.pdf), Reporting Guidelines Checklists. Additional File 1 includes completed checklists for the relevant reporting guidelines – Standards for Reporting Implementation Studies (StaRI) and the Template for Intervention Description and Replication (TIDieR).

## Data Availability

The datasets generated and/or analyzed during the current study are available in the Brown Digital Repository, which may be accessed at: 10.26300/jfy9-fa83.

## Abbreviations

ePCTs: Embedded pragmatic clinical trials
RCTs: Randomized clinical trials
ADRD: Alzheimer’s Disease and Related Dementias
US: United States
NHs: Nursing homes
M&M: Music & Memory
METRIcAL: Music & MEmory: A Pragmatic TRial for Nursing Home Residents With Alzheimer’s Disease
FIF: Framework for Implementation Fidelity
MDS: Minimum Data Set
CASPER: Certification and Survey Provider Enhanced Reporting
ADL: Activities of Daily Living
CMS: Centers for Medicare & Medicaid Services
PROVEN: Pragmatic Trial of Video Education in Nursing Homes
