# Supplementary material for: Measuring implementation fidelity in a cluster-randomized pragmatic trial: development and use of a quantitative multi-component approach
Source: Trials. 2022 Jan 15;23:43. doi: 10.1186/s13063-022-06002-8 (PMC8761354; doi:10.1186/s13063-022-06002-8)
Supplement: Supplementary file 1 — Additional file 1. Portable Document Format (.pdf), Reporting Guidelines Checklists. Additional File 1 includes completed checklists for the relevant reporting guidelines – Standards for Reporting Implementation Studies (StaRI) and the Template for Intervention Description and Replication (TIDieR). [file 13063_2022_6002_MOESM1_ESM.pdf]

Standards for Reporting Implementation Studies: the StaRI checklist for completion

The StaRI standard should be referenced as: Pinnock H, Barwick M, Carpenter C, Eldridge S, Grandes G, Griffiths CJ, Rycroft-Malone J, Meissner P, Murray E, Patel A, Sheikh A, Taylor SJC for the StaRI Group. Standards for Reporting Implementation Studies (StaRI) statement. *BMJ* 2017;356:i6795

The detailed Explanation and Elaboration document, which provides the rationale and exemplar text for all these items is: Pinnock H, Barwick M, Carpenter C, Eldridge S, Grandes G, Griffiths C, Rycroft-Malone J, Meissner P, Murray E, Patel A, Sheikh A, Taylor S, for the StaRI group. Standards for Reporting Implementation Studies (StaRI). Explanation and Elaboration document. *BMJ Open* 2017 2017;7:e013318

Notes: A key concept of the StaRI standards is the dual strands of describing, on the one hand, the implementation strategy and, on the other, the clinical, healthcare, or public health intervention that is being implemented. These strands are represented as two columns in the checklist.

The primary focus of implementation science is the implementation strategy (column 1) and the expectation is that this will always be completed.	The evidence about the impact of the intervention on the targeted population should always be considered (column 2) and either health outcomes reported or robust evidence cited to support a known beneficial effect of the intervention on the health of individuals or populations.

The StaRI standards refers to the broad range of study designs employed in implementation science. Authors should refer to other reporting standards for advice on reporting specific methodological features. Conversely, whilst all items are worthy of consideration, not all items will be applicable to, or feasible within every study.

Checklist item		Reported on page #	Implementation Strategy	Reported on page #	Intervention
			"Implementation strategy" refers to how the intervention was implemented		"Intervention" refers to the healthcare or public health intervention that is being implemented.
**Title and abstract**					
Title	**1**	1	Identification as an implementation study, and description of the methodology in the title and/or keywords		
Abstract	**2**	4-5	Identification as an implementation study, including a description of the implementation strategy to be tested, the evidence-based intervention being implemented, and defining the key implementation and health outcomes.		
**Introduction**					
Introduction	**3**	6	Description of the problem, challenge or deficiency in healthcare or public health that the intervention being implemented aims to address.		
Rationale	**4**	7	The scientific background and rationale for the implementation strategthe y (including any	7	The scientific background and rationale for the intervention being implemented (including evidence

			underpinning theory/framework/model, how it is expected to achieve its effects and any pilot work).		about its effectiveness and how it is expected to achieve its effects).
Aims and objectives	**5**	8	The aims of the study, differentiating between implementation objectives and any intervention objectives.		
**Methods: description**					
Design	**6**	9	The design and key features of the evaluation, (cross referencing to any appropriate methodology reporting standards) and any changes to study protocol, with reasons		
Context	**7**	8	The context in which the intervention was implemented. (Consider social, economic, policy, healthcare, organisational barriers and facilitators that might influence implementation elsewhere).		
Targeted 'sites'	**8**	8,17	The characteristics of the targeted 'site(s)' (e.g locations/personnel/resources etc.) for implementation and any eligibility criteria.	8	The population targeted by the intervention and any eligibility criteria.
Description	**9**	9-10	A description of the implementation strategy	7	A description of the intervention
Sub-groups	**10**	N/A	Any sub-groups recruited for additional research tasks, and/or nested studies are described		
**Methods: evaluation**					
Outcomes	**11**	11-15	Defined pre-specified primary and other outcome(s) of the implementation strategy, and how they were assessed. Document any pre-determined targets	11-15	Defined pre-specified primary and other outcome(s) of the intervention (if assessed), and how they were assessed. Document any pre-determined targets
Process evaluation	**12**	9	Process evaluation objectives and outcomes related to the mechanism by which the strategy is expected to work		
Economic evaluation	**13**	N/A	Methods for resource use, costs, economic outcomes and analysis for the implementation strategy	N/A	Methods for resource use, costs, economic outcomes and analysis for the intervention
Sample size	**14**	8	Rationale for sample sizes (including sample size calculations, budgetary constraints, practical considerations, data saturation, as appropriate)		
Analysis	**15**	15-16	Methods of analysis (with reasons for that choice)		

**Results**					
Characteristics	**17**		Proportion recruited and characteristics of the recipient population for the implementation strategy		Proportion recruited and characteristics (if appropriate) of the recipient population for the intervention
Outcomes	**18**	17-22	Primary and other outcome(s) of the implementation strategy	17-22	Primary and other outcome(s) of the Intervention (if assessed)
Process outcomes	**19**	18-20	Process data related to the implementation strategy mapped to the mechanism by which the strategy is expected to work		
Economic evaluation	**20**	N/A	Resource use, costs, economic outcomes and analysis for the implementation strategy	N/A	Resource use, costs, economic outcomes and analysis for the intervention
Sub-group analyses	**21**	N/A	Representativeness and outcomes of subgroups including those recruited to specific research tasks		
Fidelity/ adaptation	**22**	19-20	Fidelity to implementation strategy as planned and adaptation to suit context and preferences	17-18	Fidelity to delivering the core components of intervention (where measured)
Contextual changes	**23**	N/A	Contextual changes (if any) which may have affected outcomes		
Harms	**24**	N/A	All important harms or unintended effects in each group		
**Discussion**					
Structured discussion	**25**	23-25	Summary of findings, strengths and limitations, comparisons with other studies, conclusions and implications		
Implications	**26**	23	Discussion of policy, practice and/or research implications of the implementation strategy (specifically including scalability)	N/A	Discussion of policy, practice and/or research implications of the intervention (specifically including sustainability)
**General**					
Statements	**27**	27-28	Include statement(s) on regulatory approvals (including, as appropriate, ethical approval, confidential use of routine data, governance approval), trial/study registration (availability of protocol), funding and conflicts of interest		

TIDieR
Template for Intervention Description and Replication

The TIDieR (Template for Intervention Description and Replication) Checklist*:

Information to include when describing an intervention and the location of the information

Item number	Item	Where located ** Primary paper (page or appendix number)	Other † (details)
	**BRIEF NAME**		
**1.**	Provide the name or a phrase that describes the intervention.	7	
	**WHY**		
**2.**	Describe any rationale, theory, or goal of the elements essential to the intervention.	7, 10	Study Protocol
	**WHAT**		
**3.**	Materials: Describe any physical or informational materials used in the intervention, including those provided to participants or used in intervention delivery or in training of intervention providers. Provide information on where the materials can be accessed (e.g. online appendix, URL).	10	
**4.**	Procedures: Describe each of the procedures, activities, and/or processes used in the intervention, including any enabling or support activities.	7, 8, 13, 14	
	**WHO PROVIDED**		
**5.**	For each category of intervention provider (e.g. psychologist, nursing assistant), describe their expertise, background and any specific training given.	10	
	**HOW**		
**6.**	Describe the modes of delivery (e.g. face-to-face or by some other mechanism, such as internet or telephone) of the intervention and whether it was provided individually or in a group.	10	
	**WHERE**		
**7.**	Describe the type(s) of location(s) where the intervention occurred, including any necessary infrastructure or relevant features.	8	

	**WHEN and HOW MUCH**		
**8.**	Describe the number of times the intervention was delivered and over what period of time including the number of sessions, their schedule, and their duration, intensity or dose.	12	
	**TAILORING**		
**9.**	If the intervention was planned to be personalised, titrated or adapted, then describe what, why, when, and how.	10	
	**MODIFICATIONS**		
**10.‡**	If the intervention was modified during the course of the study, describe the changes (what, why, when, and how).	N/A	
	**HOW WELL**		
**11.**	Planned: If intervention adherence or fidelity was assessed, describe how and by whom, and if any strategies were used to maintain or improve fidelity, describe them.	13, 14, 15	
**12.‡**	Actual: If intervention adherence or fidelity was assessed, describe the extent to which the intervention was delivered as planned.	18, 19, 20	

** **Authors** - use N/A if an item is not applicable for the intervention being described. **Reviewers** – use '?' if information about the element is not reported/not sufficiently reported.

† If the information is not provided in the primary paper, give details of where this information is available. This may include locations such as a published protocol or other published papers (provide citation details) or a website (provide the URL).

‡ If completing the TIDieR checklist for a protocol, these items are not relevant to the protocol and cannot be described until the study is complete.

* We strongly recommend using this checklist in conjunction with the TIDieR guide (see *BMJ* 2014;348:g1687) which contains an explanation and elaboration for each item.

* The focus of TIDieR is on reporting details of the intervention elements (and where relevant, comparison elements) of a study. Other elements and methodological features of studies are covered by other reporting statements and checklists and have not been duplicated as part of the TIDieR checklist. When a **randomised trial** is being reported, the TIDieR checklist should be used in conjunction with the CONSORT statement (see www.consort-statement.org) as an extension of **Item 5 of the CONSORT 2010 Statement.** When a **clinical trial protocol** is being reported, the TIDieR checklist should be used in conjunction with the SPIRIT statement as an extension of **Item 11 of the SPIRIT 2013 Statement** (see www.spirit-statement.org). For alternate study designs, TIDieR can be used in conjunction with the appropriate checklist for that study design (see www.equator-network.org).
